# Isolation of Rice Bran Lectins and Characterization of Their Unique Behavior in Caco-2 Cells

**DOI:** 10.3390/ijms18051052

**Published:** 2017-05-13

**Authors:** Hajime Nakata, Ching Yu Lin, Maryam Abolhassani, Tomohisa Ogawa, Hiroaki Tateno, Jun Hirabayashi, Koji Muramoto

**Affiliations:** 1 Graduate School of Life Sciences, Tohoku University, Katahira 2-1-1, Aoba-ku, Sendai 980-8577, Japan; Hajime.Nakata@sapporobeer.co.jp (H.N.); halumi0121@hotmail.com (C.Y.L.); maryam@biochem.tohoku.ac.jp (M.A.); ogawa@biochem.tohoku.ac.jp (T.O.); 2 National Institute of Advanced Industrial Science and Technology, 1-1-1 Umezono, Ibaraki 305-8568, Japan; h-tateno@aist.go.jp (H.T.); jun-hirabayashi@aist.go.jp (J.H.)

**Keywords:** *Oryza sativa*, lectin, rice bran lectin, Caco-2 cells

## Abstract

Rice bran lectins, named as RBA1 and RBA2, were isolated from *Oryza sativa* in two chromatography steps: affinity chromatography and cation-exchange chromatography. RBA1 was found to be composed of a covalently linked heterodimer of 20- and 12-kDa subunits, and RBA2 was a noncovalently linked dimer of 12-kDa subunits. Both RBA1 and RBA2 bound to desialylated complex glycoproteins such as fetuin, α1-acid glycoprotein, and transferrin, and agalactosylated complex glycoproteins such as agalacto fetuin, agalacto-α1-acid glycoprotein, and agalacto-transferrin, in addition to chitooligosacchrides. RBAs were heat stable up to 80 °C and stable at pH 4–10. RBA1 increased the transport of the fluorescent marker, rhodamine 123, which is known to be transported via the P-glycoprotein-mediated efflux pathway across human intestinal Caco-2 cell monolayers. Furthermore, RBA1 itself was transported to the basolateral side of the monolayers via an endocytotic pathway.

## 1. Introduction

Lectins are a class of proteins that recognize and specifically bind carbohydrates, and are found in a wide variety of plants, animals, and microorganisms [1]. Currently, a few hundred plant lectins have been isolated and characterized in terms of their molecular structures, carbohydrate specificities, and biological properties [2]. The ubiquitous presence of lectins means that foods and foodstuffs contain varying amounts of lectins. In particular, foodstuffs of plant origin, such as legumes, fruits, tubers and seeds like cereals, typically contain high levels of lectins. Because many lectins, such as legume lectins, are relatively stable against heat denaturation and proteolytic digestion, the digestive tract is constantly exposed to the bioactive lectins contained in foods and feed [3]. Thus lectins interact with the epithelial surface of the intestine and cause physiological effects in humans and animals, particularly when consumed in large quantities. The modulating effects of lectins on the transport system of human intestinal Caco-2 cell monolayers have been demonstrated using fluorescent markers, including lucifer yellow (LY) for the paracellular pathway, fluorescein (FL) for the monocarboxylic acid transport pathway, rhodamine 123 (RH) for the P-glycoprotein-mediated efflux pathway, and calcein (CA) for the multidrug resistance associated protein-mediated efflux pathway [4,5,6,7]. For example, the transport of FL was increased by soybean lectin (SBA), rice bran lectin (RBA), and wheat germ lectin (WGA). The transport of LY was increased by WGA and *Aspergillus oryzae* lectin (AOL). The transport of RH was increased by RBA, WGA, and AOL. These results suggest that lectins affect the absorption of food factors transported by various pathways.

RBA was first isolated by a combination of affinity chromatography with ovomucoid-Sepharose and cation-exchange chromatography with CM-cellulose [8]. The lectin was a dimeric protein composed of two 19-kDa subunits. The 19-kDa subunit gave 8- and 11-kDa subunit after reducing treatment. Since then, RBAs with different molecular masses have been isolated from both rice bran and flour [9]. For example, Tabary et al. [10] reported that RBA was 36 kDa and composed of two non-identical disulfide-linked subunits of 19 and 15 kDa. These contradictory variable molecular masses were thought to be related to the presence of protease-sensitive sites in the lectin. Wilkins and Raikhel [11] isolated two cDNA clones encoding RBA and investigated their expression at the molecular and cellular levels. The cDNA clones coded for an identical 23-kDa protein which was processed to the mature polypeptide of 18 kDa by co-translational cleavage of a 2.6-kDa signal sequence and selective removal of a 2.7-kDa C-terminal peptide. The 18-kDa subunit underwent a proteolytic cleavage event to yield two subunits of 8 and 10 kDa. RBA is specific for *N*-acetylglucosamine (GlcNAc) and its oligomers, and is classified into the chitin-binding lectin family as a WGA, which is a homodimer of 19-kDa subunit. It has been shown that RBA is mitogenic against mouse splenic lymphocytes and induces apoptosis associated with cell cycle arrest in human monoblastic leukemia U937 cells [12]. However, the biochemical properties of RBA that affect its utility as a food factor or biochemical tool have not been investigated.

In this study, we isolated lectins from rice bran and characterized their biochemical properties in more detail to evaluate lectins as food factors and biochemical tools. The lectins, named as RBA1 and RBA2, showed characteristic sugar binding specificity. Moreover, RBA1 exhibited a unique behavior in human intestinal Caco-2 cells.

## 2. Results

### 2.1. Isolation of Rice Bran Lectins (RBAs)

**Figure 1:**
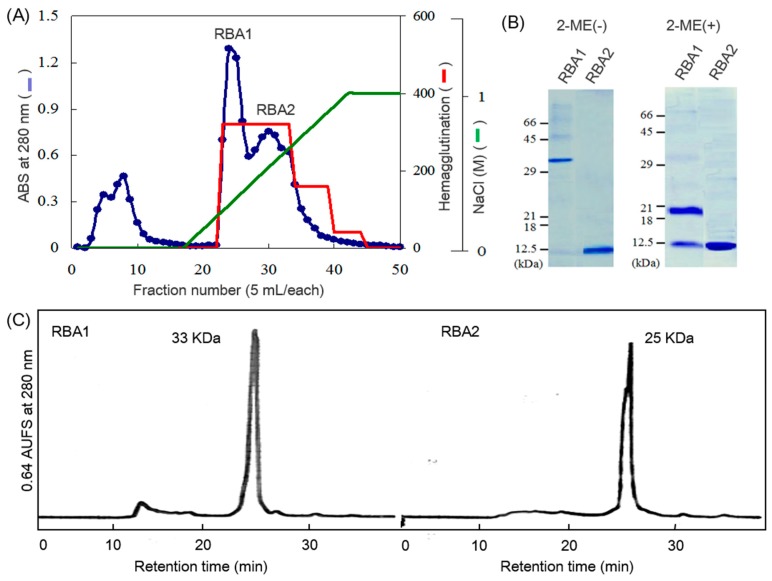
Purification of the rice bran lectins (RBAs). (**A**) The RBAs obtained by affinity chromatography were separated by ion exchange chromatography on a CM-cellulofine column equilibrated with 50 mM sodium acetate (pH 6.0) and eluted with a linear gradient of NaCl (0–1.0 M) in the same buffer. Peaks 1 and 2 were collected and designated as RBA1 and RBA2, respectively; (**B**) Sodium dodecyl sulfate-polyacrylamide gel electrophoresis (SDS-PAGE) patterns of RBAs on 15% acrylamide gels; (**C**) Size exclusion chromatography of RBAs on a PC300S column (4.6 × 250 mm) in 50 mM sodium phosphate (pH 6.9) containing 0.25 M NaCl. Flow rate was 0.2 mL/min.

Lectin fraction was isolated from defatted rice bran by affinity chromatography on a chitin column and ion-exchange chromatography on a carboxymethyl (CM)-cellulofine column (Figure 1A). The fractions exhibiting hemagglutination activity were collected separately, dialyzed against water and lyophilized to yield rice bran lectins, named as RBA1 and RBA2. They showed single bands at 32 and 12 kDa, respectively, when analyzed by sodium dodecyl sulfate-polyacrylamide gel electrophoresis (SDS-PAGE) under non-reducing conditions. The 32-kDa protein representing RBA1 was split into 20- and 12-kDa proteins under reducing conditions (Figure 1B). The recoveries of RBA1 and RBA2 were both 14% after purification by cation exchange chromatography (Table 1).

RBAs separated by SDS-PAGE were electroblotted onto a polyvinylidene difluoride (PVDF)-membrane to analyze their N-terminal amino acid sequences with a gas-phase protein sequencer. The obtained sequences were compared with those of other plant proteins by using the BLAST and FASTA programs from the NCBI. The N-terminal amino acid sequence of 12-kDa protein of RBA1 was determined to be GELCPNNMCCSQWGYCGLGSEFCGNGCQ, which was identical to that of *Oryza sativa* “Japonica” lectin. RBA2 showed the same N-terminal amino acid sequence. The sequencing of 20-kDa protein of RBA1 could not be achieved due to its blocked N-terminus.

The molecular masses of RBA1 and RBA2 were estmated to be 33 and 25 kDa, respectively, by size exclusion chromatography on a column with immobilized phoshorylcholine [13]. These results showed that RBA1 assembled as a covalently linked heterodimer of 20- and 12-kDa subunits, and RBA2 assembled as a noncovalently linked homodimer of 12-kDa subunits.

### 2.2. Hemagglutination Activity of RBAs

The minimum concentrations of RBA1 and RBA2 required to express hemagglutination activity against rabbit erythrocytes were 0.1 and 1.5 µg/mL, respectively. RBA1 lost 50% of its activity after heating at 90 °C for 30 min, whereas RBA2 lost 90% of its activity after treatment at 90 °C for 30 min. Both RBA1 and RBA2 completely lost their activities after heating at 100 °C for 30 min. RBAs were stable at pH 4–10. RBA1 lost 50% of its activity at pH 2 and RBA2 lost 70% of its activity under the same condition. RBAs did not lose their activities following ethylenediaminetetraacetic acid (EDTA) treatment, indicating that they did not require divalent cations for activity.

### 2.3. Sugar Binding Specificity

RBA1 and RBA2 showed similar sugar-binding specificities; among the saccharides tested, chitooligosacchrides strongly inhibited the RBAs. Particularly, penta-*N*-acetylchitopentaose was the strongest inhibitor; the lowest concentrations used to achieve an inhibitory effect for RBA1 and RBA2 were 0.02 and 0.04 mM, respectively (Table 2). Interestingly, the hemagglutination activity of RBA2 but not RBA1 was inhibited by rather high concentrations of D-glucosamine and D-galactosamine.

**Figure 2:**
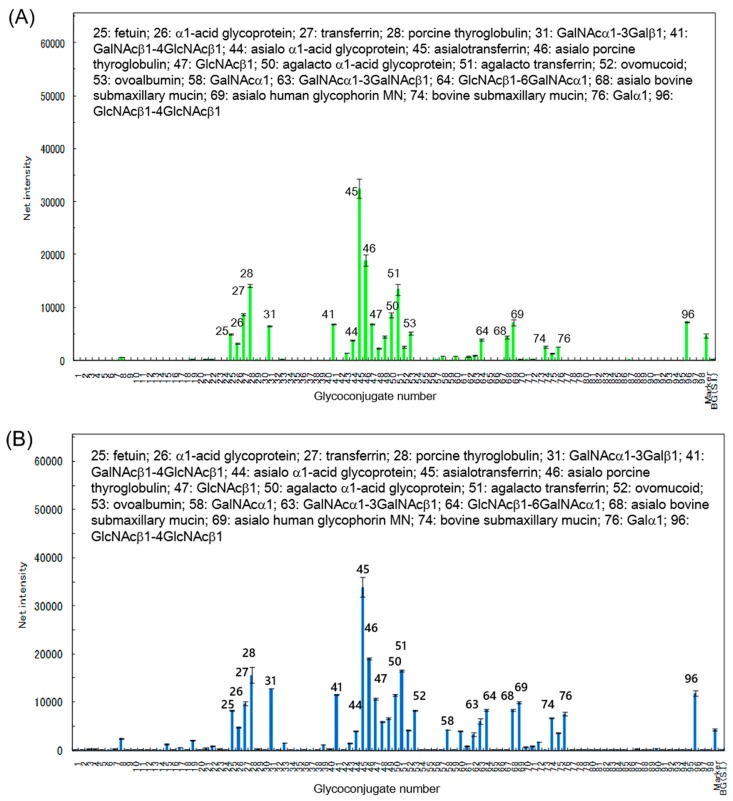
Specificity profiling of RBAs analyzed by glycoconjugate micoarray. Scan images of RBAs (12 ng/well) were analyzed with the Array Pro analyzer version 4.5. The net intensity value for each spot was determined as the signal intensity minus the background value. Data are the average ± standard deviation (S.D.) of triplicate determinations. Detailed information of the glycans can be found in reference [14]. (**A**) RBA1; (**B**) RBA2.

The carbohydrate-binding specificity of RBAs was investigated using glycoconjugate microarray assays in which 96 glycoconjugates were analyzed simultaneously. The fluorescent image data analyzed using Array Pro analyzer Ver. 4.5 (Media Cybernetics, Inc., Rockville, MD, USA), are shown in Figure 2. The RBAs showed similar affinity profiles. They bound to desialylated complex glycoproteins, such as futuin, α1-acid glycoprotein, and transferrin, as well as to agalactosylated complex glycoproteins such as agalacto-fetuin, agalacto-α1-acid glycoprotein, and agalacto-transferrin, indicating that they preferentially bound to the *N*-linked glycoproteins that were desialylated or agalactosylated. In addition, RBAs bound to LacDiNAc (*N*-acetylgalactosamine β-(1→4) *N*-acetylglucosamine), α-GalNAc (α-*N*-acetylgalactosamine) such as bovine submaxillary mucin, and β-GlcNAc (β-*N*-acetylglucosamine). These results are in good agreement with those of the sugar inhibition assay, in that RBAs showed high affinity against GlcNAc at the nonreducing end of oligosaccharides.

### 2.4. Transport of Fluorescein Isothiocyanate-I (FITC)-Labeled Lectins

RBA1, together with another lectin, was used to investigate the behavior in Caco-2 cells. RBA1, AOL, WGA, and Japanese jack bean lectin (CGA) were fluorescently labeled with fluorescein isothiocyanate-I (FITC). The FITC-labeled lectins were quantified by fluorescence intensity measurements or by enzyme-linked immunosorbent assay (ELISA) using an anti-fluorescein antibody. Differentiated Caco-2 cells were incubated with FITC-labeled lectins for 4 h to examine their cytotoxic activity against the cells. None of the lectins used showed cytotoxicity even at 200 µg/mL as assessed by the WST assay. Similarly, none of endocytosis inhibitors, amiloride, nystatin, or chlorpromazine showed cytotoxicity against Caco-2 cells at the concentrations evaluated in this study.

**Figure 3:**
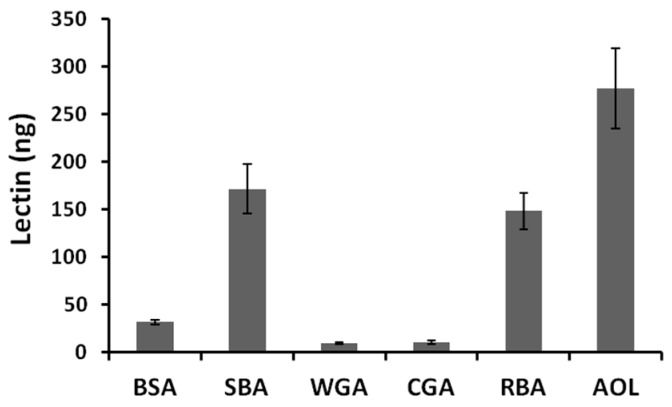
Amounts of lectins transported to the basolateral side across Caco-2 cell monolayers. BSA: bovine serum albumin; SBA: soybean lectin; WGA: wheat germ lectin; CGA: Japanese jack bean lectin; RBA: rice bran lectin 1; AOL: *Aspergillus oryzae* lectin. Each value is the mean ± S.D. of four experiments.

**Figure 4:**
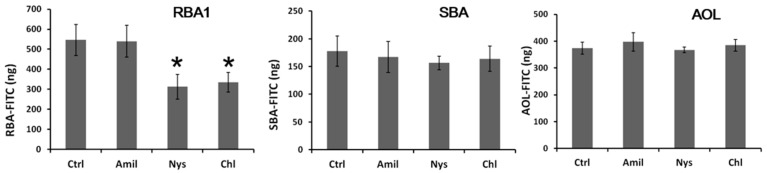
Effects of endocytosis inhibitors on the transport of lectins across Caco-2 cell monolayers. Amil: amiloride; Nys: nystatin; Chl: chlorpromazine. Each value is the mean ± S.D. of six experiments. * *p* < 0.05 compared to the control.

FITC-labeled lectins were added to the apical side of Caco-2 cell monolayers, and the lectins transported to the basolateral side were quantified indirectly via fluorescence or ELISA. The fluorescence intensity on the basolateral side increased in a time-dependent manner. The amount of SBA, RBA1, and AOL transported to the basolateral side was higher than that of bovine serum albumin (BSA) (Figure 3). Additionally, the amount of WGA and CGA transported was lower than that of BSA. Transport of SBA and AOL was not inhibited by the endocytosis inhibitors. In contrast, nystatin and chlorpromazine reduced the transport of RBA1, suggesting that it was transported partly via a clathrin-mediated endocytosis pathway (Figure 4).

### 2.5. Effect of RBA1 on Caco-2 Cell Monolayers

**Figure 5:**
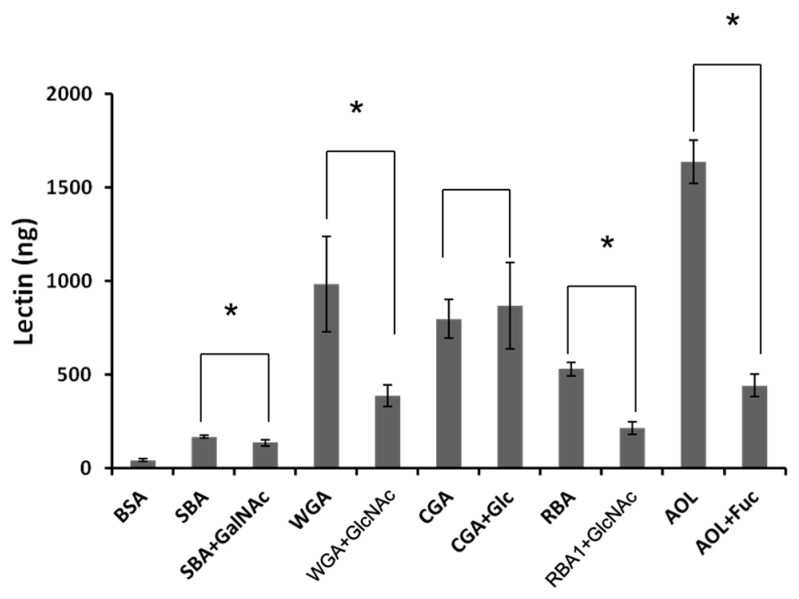
Binding of lectins to Caco-2 cell monolayers in the presence or absence of their specific sugars. Each value is the mean ± S.D. of six experiments. * *p* < 0.05 compared with the control.

**Figure 6:**
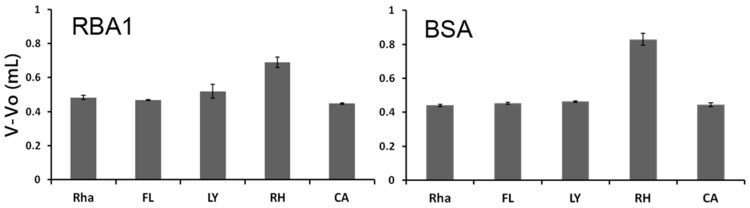
Frontal chromatography analysis of RBA1 and BSA. Interaction between RBA1 and fluorescent markers at pH 7.3 was examined. Pyridylaminated (PA)-rhamnose (Rha) was used as control. FL: fluorescein, LY: luciefer yellow, RH: rhodamine 123 and CA: calcein.

Regardless of the transport across the Caco-2 cell monolayers, all lectins bound to the monolayers (Figure 5). Except for CGA, the binding of SBA, WGA, RBA1, and AOL were inhibited by their specific sugars, GalNAc for SBA, GlcNAc for WGA and RBA1, and L-fucose for AOL, indicating that the lectins bound to specific sugar chains on Caco-2 cells. To explore the relationship between RBA1 transport and its effect on fluorescent marker transport across Caco-2 cell monolayers, the interaction of RBA1 with the markers was examined by frontal affinity chromatography (Figure 6). The interaction was evaluated by determining V − V_0_, which is the difference between the elution front volume of the fluorescent markers and that of pryridylaminated (PA)-rhamnose. RBA1 interacted strongly with RH at pH 7.3, but not with other markers. The interaction was slightly inhibited by 10 mM GlcNAc. BSA showed a similar interaction with RH. In contrast, the interaction of the proteins with FL or LY was very weak and the interaction was observed under acidic conditions.

**Figure 7:**
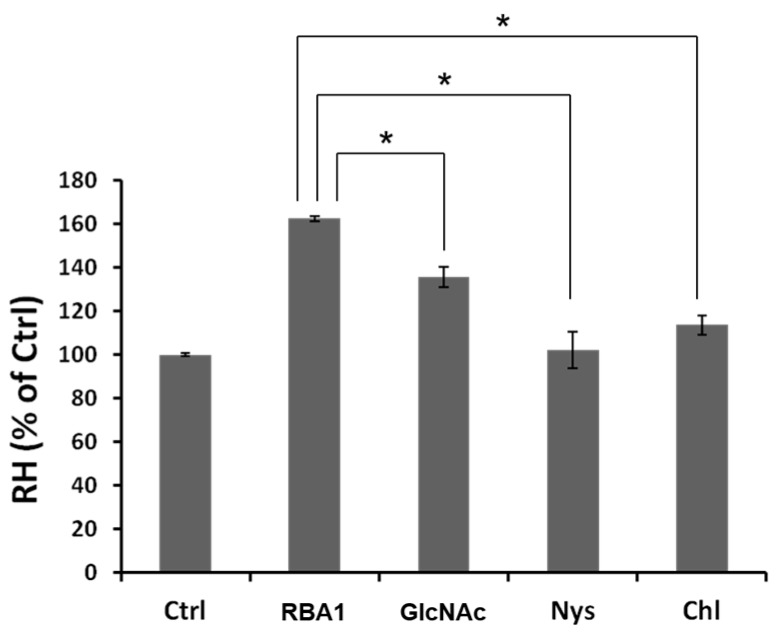
Amounts of RH transported across Caco-2 cell monolayers. Each value is the mean ± S.D. of four experiments. * *p* < 0.05 compared to control.

The transport of RH across Caco-2 cell monolayers was increased by 162% by the addition of RBA1 (Figure 7). Treatment with nystatin or chlorpromazine abolished this effect. The addition of 10 mM GlcNAc also decreased the transport.

## 3. Discussion

Rice bran is an underutilized co-product of rice milling and comprises approximately 10% by weight of rough rice. It is rich in protein, fat, carbohydrate, minerals, and vitamins. Rice bran also contains various bioactive molecules such as γ-oryzanol and lectins. Although its usefulness and nutritional potential are well known, it is largely discarded or used as animal feed or fertilizer [15]. Numerous recent studies have revealed that lectins from foodstuffs or traditional herbal medicines have many interesting biological functions including immunomodulating effects [16], selective cytotoxicity against cancer cells [17], anti-microbial and insecticidal activity [18], and modulating effects on the intestinal transport system [7]. In addition, many lectins are currently used as important tools in the fields of biochemistry, cell biology and immunology, as well as for diagnostic and physiologic purposes in cancer research; furthermore, they have been used as therapeutic agents in clinical trials [19]. In this study, RBA1 and RBA2 were isolated from rice bran by affinity and cation exchange chromatography and their biochemical properties were investigated.

Like other cereal lectins, RBA exhibits molecular masses around 30–38,000, however, in contrast to WGA, it is not dissociable at acidic pH and is dissociated into subunits by reducing agent [10]. The amino acid sequence of RBA exhibits 73% identity with that of WGA within the coding region of the mature subunits [10]. The essential difference between the molecular structures of RBA and WGA lies in the proteolytic cleavage of the subunit in RBA but not in WGA, which might produce the contradictory variable molecular masses of RBA subunits reported so far. The 18-kDa subunits (173 amino acid residues) of RBA, which are typically cleaved between residues 94 and 95 into smaller polypeptides of 10 and 8 kDa, corresponding to the amino- and carboxyl-terminal regions, respectively [8,20]. Each part contains two hevein domains, which are a characteristic motif of the chitin-binding lectin family. The amino-terminal residue of rice lectin is a glutamine, which generally cyclizes to a pyroglutamic acid residue that is resistant to Edman degradation. In fact, the expected amino-terminal sequence, QTCGKQN, could not be detected in any protein bands in this study. In contrast, the expression of rice lectin was investigated at the molecular and cellular levels using cDNA clones [11]. The cDNAs encoded a protein of 199 amino acids with a calculated Mr of 20,172. Cleavage of the protein between residues 94 and 95 generated polypeptides of 94 and 105 residues. Although this processing has not been confirmed, the calculated molecular masses of the polypeptides are similar to the values estimated by SDS-PAGE in this study. The molecular masses of intact RBA1 and RBA2, estimated by size exclusion chromatography, indicate that RBA1 assembled as a covalently linked heterodimer of 20- and 12-kDa subunits and RBA2 assembled as a noncovalently linked homodimer of 12-kDa subunits. Although there is no report describing such a small rice lectin as RBA2, a chitin binding lectin of similar size consisting of hevein domains has been isolated from stinging nettle (*Urtica dioica*) [21].

As other chitin-binding lectins, RBAs showed higher affinity against GlcNAc-oligomers than that of GlcNAc. The binding site of WGA shows the highest complementarity to *N*-acetylchitotriose, whereas RBAs appears to prefer longer sugar chains. RBA1 and RBA2 showed different affinity toward simple sugars such as d-glucosamine and d-galactosamine, likely because of their distinct subunit structures consisting of multiple sugar-binding sites in each subunit. In addition, contrary to WGA, RBAs exhibited no specificity toward *N*-acetylneuraminic acid as described previously [9]. Glycoconjugate microarray assays showed that RBAs bound to various asialo- and agalacto-glycoproteins. These sugar-binding properties may be related to the unique behaviors of RBA1 in Caco-2 cells.

WGA is known to be endocytosed by small intestinal epithelial cells and transcytosed into systemic circulation [22]. In addition, the transcellular transport of WGA-functionalized nanoparticles was reported to occur in a cytoskeleton-dependent manner and mainly via a clathrin-mediated mechanism [23]. However, in this study, less WGA than RBA1 was transported across Caco-2 cell monolayers, although WGA showed strong effects on the transport system of the cell monolayers [7]. We have no explanation for this phenomenon at present.

Transport of intact peptides and proteins from the intestinal lumen into the blood is a unique phenomenon compared to processes such as food digestion and absorption. Intestinal absorption of minute amounts of proteins is, however, considered a normal physiological process [24]. The transport mechanism is complex and is thought to include multiple pathways [25]. Oligopeptides can be transported by specific transporters, the paracellular pathway, and transcytosis. The paracellular pathway is a non-degradative transport route involving the tight junction (TJ) and consisting of many membrane proteins. The TJ is regulated by a variety of internal and external factors. We showed that chum salmon egg lectin increased intracellular Ca^2+^ significantly after 2 h incubation in a dose-dependent manner, resulting in depolymerization of β-actin in the cytoskeleton to cause reversible TJ structural and functional disruption without affecting the expression of TJ proteins such as claudin-1 [26]. Chum salmon egg lectin increased the TJ-mediated paracellular transport. Oligosaccharides can also be transported by transcytosis, particularly when they have affinity for the cell membrane surface [25]. The present results indicate that the binding of RBA1 to the Caco-2 cell surface enhanced adsorptive endocytosis, thus accelerating the transcellular transport. Endocytosis is divided into clathrin-mediated and clathrin-independent endocytosis. Three endocytosis inhibitors were evaluated in this study. Amiloride is a micropinocytosis inhibitor, whereas nystatin and chlorpromazine are clathrin-mediated endocytosis inhibitors [27]. The transport of SBA and AOL was not inhibited by any of these inhibitors. In contrast, RBA1 was inhibited by nystatin and chlorpromazine, but not by amiloride, suggesting that a considerable amount of RBA1 was transported via clathrin-mediated endocytosis. Furthermore, some RH was transported to the basolateral side by binding to RBA1, which functioned as a carrier.

## 4. Experimental Section

### 4.1. Materials

Rice (*Oryza sativa* “Hitomebore”) bran was obtained from a local rice shop. Chitin powder was purchased from Nacalai Tesque (Kyoto, Japan). CM-cellulofine was from Chisso (Tokyo, Japan). The human colon adenocarcinoma cell line, Caco-2, was obtained from the American Type Culture Collection (Manassas, VA, USA). Dulbecco’s modified Eagle’s medium (DMEM), non-essential amino acids (NEAA), and penicillin-streptomycin (10,000 U/mL and 10 mg/mL in 0.9% NaCl, respectively), were purchased from Sigma-Aldrich (St. Louis, MO, USA). Fetal bovine serum (FBS) was from Thermo Scientific HyClone (Logan, MO, USA). Plastic dishes and 96-well microplates were from BD Biosciences (Franklin Lakes, NJ, USA). A Transwell insert with a 0.40-µm polycarbonate membrane, 6.5 mm in diameter, was purchased from Corning Costar (Corning, NY, USA). The WST-1 assay kit was from Dojindo Laboratories (Kumamoto, Japan). FITC, luciefer yellow dilithium salt, and bovine serum albumin (BSA) were purchased from Sigma-Aldrich. Rhodamine 123, calcein AM, nystatin, and chlorpromazine hydrochloride were obtained from Molecular Probes (Eugene, OR, USA). Amilorid was from Oakwood Products (West Columbia, SC, USA). Anti-fluorescein antibody and fluorescein sodium salt were obtained from Wako Chemical (Osaka, Japan). Anti-rabbit IgG antibody was from Jackson Immuno Research Lab. (West Grove, PA, USA). Pyridylaminated (PA)-rhamnose was purchased from Takara Bio (Shiga, Japan). *Aspergillus oryzae* lectin (AOL) was kindly supplied by Gekkeikan (Kyoto, Japan). Soybean lectin (SBA), wheat germ lectin (WGA), and Japanese jack bean lectin (CGA) were isolated as previously described [7]. All other reagents used in this study were of analytical grade.

### 4.2. Isolation of Rice Bran Lectin

Rice bran was defatted by extraction with three-fold volumes of hexane for 1 h twice. Defatted rice bran (150 g) was suspended in l L of 0.15 M NaCl at pH 4.5 and stirred at 4 °C overnight. After centrifugation at 8000× *g* for 40 min at 4 °C, the protein fraction in the supernatant was precipitated by the addition of solid (NH_4_)_2_SO_4_ to 60% saturation. The precipitate was collected by centrifugation, re-dissolved in 400 mL of 10 mM Tris-HCl (pH 8.0), and applied to a chitin column (2.5 × 22.5 cm) equilibrated with the same buffer. The column was washed with 2 L of 10 mM Tris-HCl (pH 8.0) and 1 L of 5 M urea in 10 mM Tris-HCl (pH 8.0) to remove unbound substances, and the bound lectin was eluted with 0.5 M formic acid. Fractions showing significant absorption at 280 nm were collected, neutralized quickly, dialyzed against water, and lyophilized.

The lectin fraction was subjected to cation exchange chromatography on a CM-cellulofine column (1.2 × 20 cm) pre-equilibrated with 50 mM sodium acetate (pH 6.0). The column was eluted with a linear gradient of 0–1.0 M NaCl in the same buffer. Absorbance of the fractions at 280 nm was measured, and fractions exhibiting hemagglutination activity were collected separately, dialyzed against distilled water, and lyophilized. Isolated rice bran lectins were named as RBA1 and RBA2, respectively.

SDS-PAGE was carried out on 15% polyacrylamide gels in the presence or absence of 2-mercaptoethanol [28]. Sample solutions were mixed with an equivalent volume of sample buffer and heated at 95 °C for 5 min. After gel electrophoresis, proteins were stained with Coomassie Brilliant Blue R-250. BSA (66 kDa), egg white albumin (45 kDa), carbonic anhydrase (29 kDa), soybean trypsin inhibitor (21 kDa), myoglobin (18 kDa), and cytochrome C (12.5 kDa) were used as standard proteins. RBA1 and RBA2 were separated by SDS-PAGE and electroblotted onto PVDF membranes. The protein bands were subjected to N-terminal amino acid sequence analysis on a gas-phase protein sequencer (PPSQ-10, Shimadzu, Kyoto, Japan).

The molecular masses of RBAs were estimated with size exclusion chromatography by using a PC300S column of phosphorylcholine immobilized on silica gel beads (5 µm) (4.6 × 250 mm, Shiseido, Tokyo, Japan) and 50 mM sodium phosphate (pH 6.9) containing 0.25 M NaCl as the mobile phase [13].

### 4.3. Hemagglutination Assay and Inhibition Assay

The lectin activity was estimated by hemagglutination against rabbit erythrocytes. Samples (50 µL) were diluted 2-fold (*v*/*v*) in series with 50 µL of 20 mM Tris-HCl/0.15 M NaCl (pH 7.5) in 96-well microtiter plates, and mixed with 50 µL of 2% rabbit erythrocyte suspension for 10 min. The mixture was incubated at room temperature for 60 min, and then hemagglutination activity was measured visually. Hemagglutination activity was defined as the titer value of the maximum dilution showing positive agglutination of rabbit erythrocytes.

Sugar binding specificity was examined by hemagglutination inhibition assay. The saccharide solutions (25 µL) were diluted 2-fold in series on 96-well microtiter plates and incubated with 25 µL of the lectin solutions with hemagglutination titer values of 2^−3^ for 20 min. The rabbit erythrocytes suspension (2%, 50 µL) was added to the mixture and incubated for an additional 60 min. Inhibitory activities were estimated as the minimum concentration of sugar needed to cause negative hemagglutination.

The thermostability of RBAs was examined by the hemagglutination assay as described above after separate incubations at various temperatures (20–100 °C) for 30 min. The pH stability of RBAs was examined in a similar manner. Lectin solutions (hemagglutination titer values of 2^−8^) were incubated at pH 4.0–10.0 overnight at 4 °C and subjected to the hemagglutination assay after adjusting the pH to 7.5 with 1 M HCl or 1 M NaOH. The buffers used were 20 mM CH_3_COONa-HCl/0.15 M NaCl (pH 4.0–6.0), 20 mM Tris-HCl/0.15 M NaCl (pH 7.0–9.0), and 50 mM sodium tetraborate/0.15 M NaCl (pH 10.0).

To test the dependence of divalent cations on hemagglutination, RBAs were treated with 0.1 M EDTA in 20 mM Tris-HCl/0.15 M NaCl (pH 8.0) at room temperature for 2 h and then dialyzed against 0.15 M NaCl overnight at 4 °C. The lectin solution was tested for hemagglutination activity in the presence of Ca^2+^ or Mg^2+^ in 20 mM Tris-HCl/0.15 M NaCl (pH 8.0).

### 4.4. Glycoconjugate Microarray Assay

Glycoconjugate microarray assay was carried out as previously described [14]. Glycoproteins and glycoside-polyacrylamide conjugates were immobilized on a microarray-grade epoxy-coated glass slide (Schott AG, Mainz, Germany). Cy3-labeled RBAs precomplexed with Cy3-labeled antibodies in the probing buffer (25 mM Tris-HCl, pH 7.4 containing 0.8% NaCl, 1% (*v*/*v*) Triton-X, 1 mM MnCl_2_, and 1 mM CaCl_2_) were applied to each chamber of the glass slides (100 µL/well) and incubated at 20 °C overnight. Fluorescence images were immediately acquired using an evanescent-field activated fluorescence scanner (SC-Profiler, Moritex, Tokyo, Japan) under Cy3 mode. Throughout the experiments, the scanning conditions of the cooled charge-coupled device (CCD) camera, i.e., resolution (5 µm), number of integrations (4), and exposure time (200 s), were fixed. Data were analyzed with the Array Pro analyzer Ver. 4.5 software (Media Cybernetics, Inc., Rockville, MD, USA). The net intensity value for each spot was determined as the signal intensity minus the background value. Data are the average ± S.D. of triplicate determinations.

### 4.5. FITC Labeling of Lectins

Lectins (20 mg) and FITC (8 mg) were dissolved in 1.0 mL of 0.1 M of sodium carbonate buffer (pH 9.0) and incubated at room temperature for 3 h in the dark. The labeled lectins were separated from free FITC by gel filtration chromatography on a Sephadex G-25 column (5 mL) with sodium phosphate-buffered saline (PBS) (pH 7.3). BSA was also labeled with FITC by the same method.

### 4.6. Cell Culture

Caco-2 cells at passage numbers 23–35 were cultured in DMEM with 10% (*v*/*v*) FBS, penicillin-streptomycin (50 IU/mL and 50 µg/mL, respectively), and 1% (*v*/*v*) NEAA, and maintained at 37 °C in a humidified atmosphere of 5% CO_2_. The cells were sub-cultured at 70–80% confluency. Caco-2 cell monolayers were prepared by seeding into 96-well microtiter plates at a density of 1.0 × 10^5^ cells/cm^2^, and maintained for 18–21 days with the culture medium being replaced every 2–3 days. For transport experiments, Caco-2 cell monolayers were prepared by seeding on Transwell inserts in 24-well plates at a density of 1.0 × 10^5^ cells/cm^2^. The apical and basolateral compartments contained 0.1 and 0.6 mL of the culture medium, respectively. The cell monolayers were maintained for 18–21 days (culture medium was replaced every 2–3 days), and the integrity of the cell monolayers was evaluated by measuring the transepithelial electrical resistance (TER) value with a Millicell-ERS instrument (Millipore, Billerica, MA, USA). Cell monolayers with TER values of >500 Ω/cm^2^ were used for subsequent experiments.

### 4.7. Cytotoxicity Assay

FITC-labeled lectins, nystatin and chlorpromazine were dissolved in Hank’s balanced salt solution (pH 7.3) (HBSS). The cell monolayers were gently rinsed twice with HBSS, and treated with lectins (0.1–200 µg/mL), nystatin (1–25 µg/mL), or chlorpromazine (0.1–6 µg/mL) for 4 h. Amiloride was dissolved in dimethyl sulfoxide and diluted 40-fold with HBSS. The cell monolayers were gently rinsed twice with HBSS, and treated with amiloride (1–100 µg/mL). After the treatment, cytotoxicity was measured using the WST-1 assay kit.

### 4.8. Transport Assay

The cell monolayers on Transwell inserts were gently rinsed twice with pre-warmed HBSS and incubated for 30 min at 37 °C. After incubation, TER values were measured to confirm the differentiation of Caco-2 cells. After removing the apical and basolateral solution, 600 µL of HBSS was added to the basolateral side and 200 µL of HBSS containing 200 µg/mL of FITC-labeled lectins to the apical side. After incubation for 4 h at 37 °C, the basolateral solutions were used for competitive ELISA or fluorescence measurements. FITC-labeled lectins were quantitated by a fluorescence microplate reader (Gemini XPS-TON, Molecular Device, Sunnyvale, CA, USA) at an excitation wavelength of 485 nm and emission wavelength of 538 nm.

For endocytosis inhibition experiments, 200 µL of HBSS containing 200 µg/mL of FITC labeled lectins was added to the apical side with100 µg/mL of amiloride, 25 µg/mL of nystatin or 6 µg/mL of chlorpromazine. After incubation for 4 h at 37 °C, the basolateral solutions were used to quantif FITC-labeled lectins.

For RH transport experiments, 200 µL of HBSS containing 50 µM of RH, 200 µg/mL of RBA and each concentration of endocytosis inhibitory reagents or 50 mM of GlcNAc was added to the apical side. After incubation for 2 h at 37 °C, the basolateral solutions were subjected to fluorescence measurement at an excitation wavelength of 485 nm and emission wavelength of 538 nm.

### 4.9. Binding of Lectins with Caco-2 Cells

The cell monolayers were gently rinsed twice with HBSS and incubated with 20 µg of FITC-lectins in 100 µL of HBSS in the presence or absence of 50 mM of specific saccharides. After incubation for 30 min at 37 °C, the cell monolayers were gently rinsed with HBSS twice and lysed with 100 µL of 0.5% SDS in PBS. The lysed cells were centrifuged at 15,000× *g* for 1 min at 4 °C, and the supernatants were subjected to fluorescence measurement at an excitation wavelength of 485 nm and emission wavelength of 538 nm.

### 4.10. Competitive ELISA

Microtiter plates were coated with each lectin labeled with FITC (5 µg/mL in 0.1 M sodium bicarbonate, pH 9.6) by incubation at 37 °C for 2 h. Excess lectins were removed by washing with PBS-T four times. The plates were incubated for 2 h with 50 µL of sample solutions and 50 µL of anti-FITC antibody (1:10,000 dilution) in PBS containing 0.5% BSA. After washing with PBS-T, the plates were reacted with 100 µL of horseradish peroxidase-conjugated goat anti-rabbit IgG (1:10,000 dilution). After washing the plates, the color was developed by the addition of *o*-phenylenediamine as a chromogen. Absorbance was measured by a plate-reader at 405 nm.

### 4.11. Frontal Affinity Chromatography

The interaction of lectins with fluorescent markers (FL, LY, RH and CA) was analyzed by frontal affinity chromatography [29]. Lectins were dissolved in coupling buffer (0.2 M NaHCO_3_ (pH 8.3), 0.5 M NaCl) and immobilized onto Hi-Trap *N*-hydroxysuccinimide-activated matrix (GE Healthcare, Little Chalfont, UK) according to the manufacturer’s instruction. To deactivate excess active *N*-hydroxysuccinimide groups, the resin was blocked and washed with 0.5 M ethanolamine (pH 8.3) in 0.5 M NaCl and 0.1 M sodium acetate (pH 4.0) containing 0.5 M NaCl, respectively. The resin was suspended in elution buffer, EDTA-PB (140 mM NaCl, 2.7 mM KCl, 10 mM Na_2_HPO_4_, 1.76 mM KH_2_PO_4_), and packed into a stainless column (4.0 × 10 mm). The column was connected to a high-performance liquid chromatography (HPLC) system. Fluorescent markers, FL, LY, RH, and CA, were dissolved in EDTA-PB (pH 7.3) to final concentrations of 1 pM, 1 nM, 100 pM, and 1 nM, respectively. Each sample solution was applied to the lectin immobilized column through 2-mL sample loop connected to an injector at a flow rate of 0.25 mL/min at 25 °C. The elution profile of fluorescent markers was monitored with Dynamix Compare Module software (Rainin Instrument Company, Tokyo, Japan). V and V_0_ were the elution front volumes of each fluorescent marker and PA-rhamnose, respectively.

### 4.12. Data Analysis

The results are shown as a percentage of control values, and expressed as the mean ±S.D. of four to six individual determinations. The data were compared by one-way analysis of variance (ANOVA), followed by Tukey’s test. The statistical differences were considered at a probability less than 5% and marked by asterisks.

## 5. Conclusions

Thus, our results suggest that RBAs modulates the transepithelial transport system in different ways. Taken together, RBAs classified in the chitin-binding family were found to have unique biochemical properties as food factors and biochemical tools.

## Figures and Tables

**Table 1:** Purification of rice bran lectins (RBAs).

Purification step	Protein (mg)	Total activity (Unit)	Recovery (%)
Extract		20288	100
60% (NH_4_)_2_SO_4_ precipitation	90	11776	58
Affinity chromatography	44	7711	38
Ion exchange chromatography			
RBA1	21	1613	14
RBA2	17	1670	14

**Table 2:** Inhibition of hemagglutination activity of RBAs by saccharides.

Sugar ^a^	RBA1 (mM)	RBA2 (mM)	Sugar ^a^	RBA1 (mM)	RBA2 (mM)
d-Glucosamine	>200	3.1	Chitotriose	>200	>200
d-Galactosamine	>200	0.8	Di-*N*-acetylchitobiose	0.78	6.25
*N*-Acetyl d-glucosamine	25	50	Tri-*N*-acetylchitotriose	0.09	0.39
*N*-Acetyl d-galactosamine	>200	50	Tetra-*N*-acetylchitotetraose	0.04	0.04
Chitosan oligosaccharide	>200	>200	Penta-*N*-acetylchitopentaose	0.02	0.04
Chitobiose	100	>200	Hexa-*N*-acetylchitohexaose	0.04	0.04

^a^ Minimum concentration of saccharides required for complete inhibition.
